# Poly(2‐propylacrylic acid)/poly(lactic‐co‐glycolic acid) blend microparticles as a targeted antigen delivery system to direct either CD4^+^ or CD8^+^ T cell activation

**DOI:** 10.1002/btm2.10068

**Published:** 2017-07-11

**Authors:** Lirong Yang, Evelyn Bracho‐Sanchez, Lawrence P. Fernando, Jamal S. Lewis, Matthew R. Carstens, Craig L. Duvall, Benjamin G. Keselowsky

**Affiliations:** ^1^ J. Crayton Pruitt Family Dept. of Biomedical Engineering, College of Engineering University of Florida Gainesville, FL 32611; ^2^ Dept. of Biomedical Engineering Vanderbilt University Nashville, TN 37235; ^3^ Dept. of Biomedical Engineering University of California Davis, CA 95616

**Keywords:** dendritic cells, endosomal escape, immunotherapy, intracellular delivery, pH‐responsive polymer, PLGA, polymer blend, PPAA

## Abstract

Poly(lactic‐co‐glycolic acid) (PLGA) based microparticles (MPs) are widely investigated for their ability to load a range of molecules with high efficiency, including antigenic proteins, and release them in a controlled manner. Micron‐sized PLGA MPs are readily phagocytosed by antigen presenting cells, and localized to endosomes. Due to low pH and digestive enzymes, encapsulated protein cargo is largely degraded and processed in endosomes for MHC‐II loading and presentation to CD4^+^ T cells, with very little antigen delivered into the cytosol, limiting MHC‐I antigenic loading and presentation to CD8^+^ T cells. In this work, PLGA was blended with poly(2‐propylacrylic acid) (PPAA), a membrane destabilizing polymer, in order to incorporate an endosomal escape strategy into PLGA MPs as an easily fabricated platform with diverse loading capabilities, as a means to enable antigen presentation to CD8^+^ T cells. Ovalbumin (OVA)‐loaded MPs were fabricated using a water‐in‐oil double emulsion with a 0% (PLGA only), 3 and 10% PPAA composition. MPs were subsequently determined to have an average diameter of 1 µm, with high loading and a release profile characteristic of PLGA. Bone marrow derived dendritic cells (DCs) were then incubated with MPs in order to evaluate localization, processing, and presentation of ovalbumin. Endosomal escape of OVA was observed only in DC groups treated with PPAA/PLGA blends, which promoted high levels of activation of CD8^+^ OVA‐specific OT‐I T cells, compared to DCs treated with OVA‐loaded PLGA MPs which were unable activate CD8^+^ T cells. In contrast, DCs treated with OVA‐loaded PLGA MPs promoted OVA‐specific OT‐II CD4+ T cell activation, whereas PPAA incorporation into the MP blend did not permit CD4^+^ T cell activation. These studies demonstrate PLGA MP blends containing PPAA are able to provide an endosomal escape strategy for encapsulated protein antigen, enabling the targeted delivery of antigen for tunable presentation and activation of either CD4^+^ or CD8^+^ T cells.

## 1 INTRODUCTION

Biomaterials have traditionally been developed to avoid provoking chronic aggravation of the immune system.^1^ This complex network of cellular interactions and processes has evolved to provide protection against foreign invaders as well as homeostatic regulation of self to nonself. Strategies that aim to manipulate components of the immune system, particularly polymer‐based vaccines, have gained attention in recent years.^1^, ^2^, ^3^, ^4^ Polymeric microparticles (MPs) offer a distinct advantage as their intrinsic immunomodulatory properties and chemical versatility fulfill desired properties in achieving long‐term protection. Generation of protective immunity following vaccination depends on antigen availability, delivery to antigen presenting cells (APCs) and further activation of innate immunity through the appropriate pathways.^5^ Current vaccination strategies are adept at generating antibodies and CD4^+^ T cell mediated immunity, but approaches to activate CD8^+^ T cell pathways, necessary to combat intracellular pathogens such as malaria, HIV and cancer, are limited. Present strategies are based on live‐attenuated or inactivated pathogens that induce robust humoral and cellular immunity. However, safety concerns associated with live vectors limits their use in infants, the elderly and the immunocompromised.^6^ Conversely, recombinant antigens based on peptides hold high potential for CD8^+^ T cell vaccine development but their dependency on adjuvants remains a major hurdle to clinical translation.^7^ In this study, we develop a unique polymer blend for the fabrication of microparticles that allow for the tuning of CD4^+^ and CD8^+^ T cell responses through targeting of dendritic cells (DCs). We hypothesize incorporation of a small amount of Poly(2‐propylacrylic acid) (PPAA) into a poly(lactic‐co‐glycolic acid) (PLGA) matrix, will lead to endosomal escape triggering cross‐presentation pathway within DCs and further activation of CD8^+^ T cells.

Dendritic cells are sentinels at the forefront of immunological responses as the most efficient APCs and regulators of adaptive immunity. Dendritic cells are constantly surveying the environment and are able to uptake, process and present antigen to naïve T cells and appropriately shape the resulting T and B cell responses.^8^ T lymphocytes recognize peptide antigens presented by APCs in the context of the major histocompatibility complex molecules (MHC) Class I and II. Peptides derived from the cytosol are loaded onto MHC Class I in the lumen of the endoplasmic reticulum and transported to the cell surface by vesicles for recognition by CD8^+^ T lymphocytes. Peptides generated by the degradation of exogenous proteins in endosomes are bound to MHC Class II and presented to CD4^+^ T cells. Dendritic cells however, have the ability to process exogenous proteins and present them in the context of MHC Class I. This function, known as cross‐presentation, diversifies the ability of the immune system to generate immunity to various pathogens.^9^

Microparticles fabricated of PLGA are one of the most widely used biocompatible, biodegradable polymeric carriers with multiple application in delivery of drugs, biomolecules and genes. PLGA systems are able to efficiently load hydrophilic and hydrophobic molecules and can easily be fabricated to specific micron size ranges, efficiently phagocytosed by DCs, and allowing targeted delivery of antigen to endosomal compartments. For sustained cytosolic delivery and activation of cross‐presentation pathways an endosomal escape strategy must be employed. Several compounds have been incorporated into PLGA to allow blended formulation with improved stability, loading and delivery capacity. Poly(2‐propylacrylic acid), has the ability to reversibly switch from a hydrophilic soluble conformation at physiological pH to a hydrophobic and membrane interactive state in response to acidic pH. Functionally, PPAA‐containing systems trigger the disruption of and release from acidified endosomal compartments, effectively targeting cargo to cytosol. However, PPAA alone cannot readily support a matrix capable of encapsulating bioactive molecules. Therefore, in this study we incorporate PPAA into the PLGA matrix of MPs in order to provide cytosolic delivery of a model antigen, while maintaining robust properties of PLGA, thus accessing MHC‐I loading and allowing the tuning of CD4^+^ and CD8^+^ T cell responses.

## 2 MATERIALS AND METHODS

### 2.1 Materials

GMP grade PLGA (L/G = 50:50, Purasorb PDLG 5004A, intrinsic viscosity = 0.4 dl/g) was purchased from Corbion Purac, Netherlands. Poly(2‐propylacrylic acid) (Mn = 20–80 kDa) and albumin from chicken egg white (Ovalbumin, OVA, 98%, Grade V, molecular weight = 44,287 Da) was obtained from Sigma‐Aldrich Co., St Louis, MO. Methylene Chloride (MC) (99.9%), N,N‐Dimethylformamide (DMF, 99.5%), bovine serum albumin, and ethylenediamine tetraacetate acid were obtained from Fisher Scientific, Fair Lawn, NJ. Polyvinyl alcohol (PVA, average MW = 15,000 g/mol, 88.63% hydrolyzed) was supplied by MP Biomedicals LLC, Solon, OH. Mounting medium for fluorescence with DAPI (sc‐24941) was obtained from Santa Cruz Biotechnology Inc., CA. 4% paraformaldehyde solution in PBS (4% PFA) and 5‐(and 6)‐carboxyfluorescein diacetate *N*‐succinimidyl ester (CFSE) were all obtained from Affymetrix Inc, Cleveland, OH. Endotoxin levels were measured using the ChromoLAL assay kit purchased from Associates of Cape Cod, Inc., Falmouth, MA.

Dulbecco's Modified Eagle Medium: Nutrient Mixture F‐12 (DMEM/F12), fetal bovine serum (FBS), and Phosphate‐buffered saline (PBS) were purchased from GE Healthcare Life Sciences, Logan, UT. l‐glutamine, sodium pyruvate, nonessential amino acids (NEAA), and ACK lysis buffer were obtained from Lonza, Walkersville, MD. Penicillin‐streptomycin solution (100×) was from Mediatech Inc, Manassas, VA. Granulocyte macrophage colony‐stimulating factor (GM‐CSF) was supplied by R&D Systems, Minneapolis, MN. MACS LS columns, mouse CD8a+ T cell isolation kit, and mouse CD4+ T cell isolation kit were obtained from Miltenyi Biotec, Auburn, Canada. Molecular probes live/dead fixable Near‐IR dead cell stain kit and DQ ovalbumin were purchased from Life Technologies. Chicken egg albumin peptides OVA 257–264 and OVA 323–339 were supplied by InvivoGen, San Diego, CA. BD Cytofix fixation buffer, purified rat antimouse CD16/CD32 (mouse BD Fc block), APC antimouse CD4, and APC rat antimouse CD8a were all from BD Biosciences, San Jose, CA.

### 2.2 Preparation of OVA‐loaded PLGA‐based microparticles

OVA‐encapsulated PLGA MPs were prepared using a modified double emulsion water‐oil‐water (w/o/w) solvent extraction technique.^10^ Briefly, 100 mg of PLGA polymer was dissolved in methylene chloride at 5% w/v ratio. 1 mg of OVA in PBS was added to 5% PLGA solution and vigorously mixed for 120 s at 35,000 rpm using a homogenizer (Fisher Scientific, NJ) to form a primary o/w emulsion. The emulsified solution was then loaded into 4 ml of 5% (w/v) PVA in water and homogenized for an additional 60 s at 35,000 rpm to form the second emulsion. Finally, the w/o/w emulsion was poured slowly in 100 ml of 1% PVA in agitation to form microparticles. The solution remained in agitation with a magnetic stirrer overnight at room temperature to evaporate methylene chloride and stabilize microparticles. The remaining solution was centrifuged at 10,000 rpm for 15 min to collect fabricated MPs and subsequently rinsed three times with PBS to remove uncaptured OVA. Collected MPs were lyophilized in a freeze‐dryer for 2 days and stored at −20°C for later use.

PPAA/PLGA blend MPs were fabricated with a modified protocol. PPAA (100 mg) were dissolved in 2 ml of DMF while 90 mg of PLGA were dissolved in 1.8 ml of MC for 10% PPAA/PLGA blend or 97 mg in 1.94 ml for 3% PPAA/PLGA blend. After polymer dissolution, 10 mg of PPAA (200 μl of PPAA solution in DMF) was added to 90 mg of PLGA in MC solution or 3 mg of PPAA (60 μl of PPAA solution in DMF) was added to 97 mg of PLGA in MC solution. In other words, 100 mg of PLGA‐PPAA (w/w) blend polymer was dissolved in a total of 2 ml of organic solvent methylene chloride/DMF. One milligram of OVA in PBS was added to 5% PLGA‐PPAA blend solution and vigorously mixed for 105 s (for 3% PPAA) or 75 s (for 10% PPAA) at 35,000 rpm to form a primary emulsion. The emulsified solution was loaded into 4 ml of 5% (w/v) PVA, pH 6.0 and re‐emulsified for additional 45 s (for 3% PPAA) or 15 s (for 10% PPAA) at 35,000 rpm. The second emulsion was poured slowly in 100 ml of 1% PVA, pH 6.0 in agitation to form microparticles.

Prior to fabrication, glassware used during process was heated to 250°C for 4 hr to minimize endotoxin contamination. Homogenizer was sterilized by submerging components in ethanol overnight and all solutions filtered sterilized. Microparticles were then tested for endotoxin content using the ChromoLAL method in accordance with manufacturer's instructions.

### 2.3 Characterization of OVA‐loaded PLGA‐based microparticles

The size and zeta potential of the different OVA‐encapsulated PLGA‐based MPs were determined using a Microtrac Nanotrac Dynamic Light Scattering Particle Analyzer (Microtrac, Montgomery, PA) and a Brookhaven ZetaPlus zeta potential analyzer (Brookhaven Instruments Corp., NY), respectively. Scanning electron microscopy (FEG‐SEM JEOL JSM—6335F, Major Analytical Instrumentation Center, University of Florida) was used to confirm size distribution and morphology of MPs. Lyophilized microparticles were suspended in DI water and deposited on glass coverslips over a double sided conductive tape attached to a metal stub. Samples were dehydrated overnight in a desiccator followed by sputter‐coating with gold/palladium before image collection at 10–15 kV.

To determine the loading efficiency of PLGA‐based MPs, 10 mg of microparticles were dissolved in 200 μl of MC followed by the addition of 1% tween 20 in PBS to disperse the protein into the aqueous phase. The suspension was centrifuged and the supernatant moved to a new tube. 200 µl of methylene chloride was added to the pellet to dissolve any remaining polymer and further encapsulated release protein. This process was repeated three times. The OVA concentration of the extracted aqueous solution was determined using a Nanodrop spectrophotometer ND1000 (ThermoScientific, Wilmington, Delaware), compared with a standard calibration curve of data obtained by series of known concentrations of OVA solution.

To evaluate the release profile of OVA from PLGA‐based MPs, 1 mg of MPs was suspended in 1 ml of PBS. Tubes were kept under agitation at 200 rpm at 37°C. At predetermined time points, MPs were centrifuged at 10,000 rpm for 10 min and 200 μl of the supernatant collected and stored at −20°C until further analysis. The medium collected was replenished with fresh PBS and particles were vortexed and placed back in the incubator. The concentration of OVA released from MPs was measured by micro BCA protein assay kit (ThermoScientific, Rockford, IL), according to manufacturer's instructions.

### 2.4 Mice

The studies reported here conform to the animal welfare ACT and the National Institutes of Health guidelines for the care and use of animals in the biomedical research. All experiments were completed in compliance with the regulations of the University of Florida Institutional Animal Care and Use Committee and in accordance with the guidelines of the Association for Assessment and Accreditation of Laboratory Animal Care. Eight to 12‐week old C57BL/6J (wild type, WT) transgenic OT‐I and OT‐II on a C57BL/6J background were purchased from the Jackson Laboratory.

### 2.5 Isolation and culture of murine bone marrow dendritic cells

Dendritic cells were derived from mouse bone marrow as previously described.^11^ In brief, mice were euthanized by CO_2_ asphyxiation followed by cervical dislocation. Tibias and femurs were harvested and marrow cells collected by flushing the shafts of the bones with a 25‐g needle using wash media (RPMI medium containing 10% heat inactivated filter sterilized fetal bovine serum, and 1% penicillin–streptomycin) and mixed to obtain a single‐cell suspension. The solution was then strained through a 70 µm cell strainer and centrifuged at 1,700 rpm for 5 min at 4°C. Erythrocytes were then removed by resuspending in ACK lysis buffer for 5 min at room temperature and centrifuging at the conditions specified above. Resulting cells were cultured in DC media (complete DMEM/F12 medium containing 2.5 mM l‐glutamine, 100 U/ml penicillin–streptomycin, 10% heat inactivated filter sterilized FBS, 1 mM sodium pyruvate, and 1x NEAA mixture and 20 ng/ml GM‐CSF). Monocytes were selected by plastic adherence for 6 days at 37°C with 95% relative humidity and 5% CO_2_. The culture was fed every 2 days by gently aspirating 50% medium and adding fresh medium. On day 6 of culture, the medium was discarded and non/loosely adherent cells containing dendritic cells were collected. A single‐cell suspension of cells was then plated immediately in round coverslips placed in 6‐well plates at 5 x 10^5^ cells/well for endosomal disruption study, or in round‐bottom 96‐well micro‐titer plates (Corning, Corning, NY) at 2.5 x 10^4^ cells/well for T cell proliferation assay.

### 2.6 Endosomal disruption by PPAA/PLGA microparticles

After overnight incubation with 100 μg of DQ‐OVA according to published methods,^12^ different PLGA‐based microparticles were washed with PBS three times to remove unbound dye and then loaded to relevant experimental groups of cultured DCs at a 3:1 ratio (MPs: cells) in DC media for 1 hr at 37°C on day 8 of DC culture. To determine the number of MPs, 0.5 mg were dissolved in 10 ml of water and counted using a hemocytometer. The cells were then washed three times with warm PBS to remove MPs that were not phagocytosed and cultured in complete DMEM/F12 medium containing GM‐CSF for 3 days. Each day, DCs were fixed with 4% PFA for 10 min at room temperature. Cell nuclei were stained with Hoechst 33342. Coverslips were then mounted on microscope slides and phase contrast microscopy of cells was performed on a Zeiss Axiovert 200 M using 40X objective lens (Carl Zeiss Microscopy, Thornwood, NY). FITC/DC area was calculated by measuring the fluorescence intensity for each channel and calculated using the AutoMeasure module within AxioVision Rel. 4.8. Briefly, parameters (intensity, brightness, area of interest) were determined using image segmentation. Then, artifacts are removed and object separation performed to determine the number of regions to be analyzed within each channel. Then, the area sum, in um^2^, of the outlined regions was determined. The area sum of the FITC channel was then divided by the area sum of the DAPI channel.

### 2.7 T cell proliferation assay by flow cytometry

On day 8 of culture, DCs were seeded with OVA‐loaded PLGA‐based microparticles at a 50:1 ratio (MPs: cells) and incubated for an additional 2 days. On day 10, CD4^+^ and CD8^+^ T cells were isolated from the spleen of OT‐I and OT‐II 8‐week old female mice. Briefly, animals were euthanized by CO_2_ asphyxiation followed by cervical dislocation. Spleens were aseptically harvested and homogenized in wash media. Cells were then passed through a 70 µm cell strainer and centrifuged at 1,700 rpm for 5 min at 4°C. Red blood cells were eliminated using ACK lysis buffer followed by centrifugation to recover lymphocytes. CD4^+^ and CD8^+^ T cells were purified using a negative selection isolation kit according to manufacturer's instructions. Resulting cells were stained with 5 µM CFSE for 10 min at room temperature to track proliferation. DCs were washed of particles three times with warm PBS and relevant groups pulsed with 100 μl of 2 μg/ml OVA peptides, 323–339 or 257–264, in DC media for 3 hr. DCs and T cells were co‐cultured for 4 days at a 1:10 ratio with a half media change on day 2. On the specified days, T cells were collected by centrifugation, washed with PBS containing 1% FBS, stained with live/dead NIR dye, and antibodies against surface markers CD4 or CD8 for 30 min at 4°C and fixed with BD Cytofix fixation buffer for 20 min at 4°C. Proliferation was measured by CFSE dilution using a Guava EasyCyte (EMD Millipore) flow cytometer. Data analysis was performed using FCS Express version 3 (De Novo Software, Los Angeles, CA).

### 2.8 Statistical analysis

Data reported were analyzed and charts were generated using Prism 5.0 (GraphPad, San Diego, CA). Statistics were done using a one‐way ANOVA followed by Tukey's post hoc analysis to make pair‐wise comparisons, with 95% confidence intervals. Two‐way ANOVA was used to determine differences in T cell proliferation with a Bonferroni's post hoc analysis. Unless otherwise indicated, data represent the mean ± *SEM*, with *p* < .05 considered statistically significant.

## 3 RESULTS

### 3.1 Microparticle characterization

**Figure 1:**
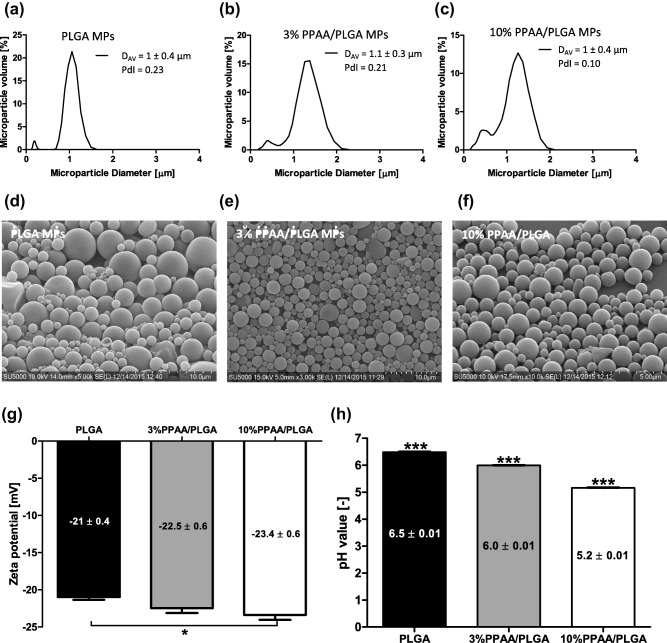
PLGA and PPAA/PLGA blend MPs characterization. Size distribution of (a) PLGA MPs, (b) 3% PPAA/PLGA MPs, (c) 10% PPAA/PLGA MPs, as determined by dynamic light scattering. Morphology of (d) PLGA MPs, (e) 3% PPAA/PLGA MPs, and (f) 10% PPAA/PLGA MPs visualized through scanning electron microscopy. Scale bar represents 10 μm (d, e) and 5 μm (f). (g) Zeta potential and (h) pH of PLGA and PPAA blend MPs were measured. The data shown represent mean ± standard error of the mean (*SEM*). Values of *p* (where *<.05 and ***<.001) were calculated using a nonparametric one‐way ANOVA with a Tukey's post‐test

**Figure 2:**
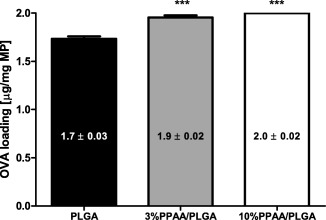
Encapsulation efficiency of PLGA and PPAA/PLGA blend MPs. Mass of ovalbumin encapsulated in each MP formulation. Data are shown as mean ± *SEM* for *n* = 3 for each microparticle formulation. Values of *p* (where ***<.001) were calculated using a nonparametric one‐way ANOVA with a Tukey's post‐test

Polymer blend MPs with fabrication compositions of 0% PPAA (PLGA only), 3% PPAA/PLGA and 10% PPAA/PLGA were loaded with ovalbumin by double emulsion solvent evaporation. Endotoxin level was tested by the ChromoLAL method against a standard curve and determined to be 0.47, 0.23, and 0.37 EU/mg for the PLGA only, 3% PPAA/PLGA, and 10% PPAA/PLGA MPs, respectively. Values are well below acceptable endotoxin levels for vaccine formulations in preclinical models.^13^ Microparticles were characterized by determining their size distribution and morphology, zeta potential, surface pH, loading and in vitro release kinetics. The size range of MPs was determined by dynamic light scattering, calculated by volume to be 1 ± 0.4 µm for the PLGA only, 1.1 ± 0.3 µm for the 3% blend, and 1 ± 0.4 µm for the 10% blend with a polydispersity index of 0.23, 0.21, and 0.10, respectively (Figure 1a–c). Scanning electron microscopy images confirmed size distribution of ∼1 µm in diameter as well as a spherical shape and smooth surfaces for all three microparticle formulations (Figure 1d–f). Zeta potential analysis revealed MPs are negatively charged under aqueous conditions with specific values of −21 ± 0.4 mV for PLGA only, −22.5 ± 0.6 mV for 3% PPAA blend, and −23.4 ± 0.6 mV for the 10% PPAA blend (Figure 1g). The negative surface charge increased modestly, in proportion to the percentage of PPAA in the formulation of PPAA/PLGA blend microparticles, which suggested that PPAA was partly present on the surface and increasing negativity was due to deprotonated carboxyl of PPAA. All formulations showed effective loading, with PLGA only MPs encapsulating 1.7 ± 0.03 μg OVA/mg MP, 3% PPAA/PLGA MPs 1.9 ± 0.02 μg/mg MP, and 10% PPAA/PLGA MPs 2.00 ± 0.02 μg/mg MP, respectively (Figure 2).

**Figure 3:**
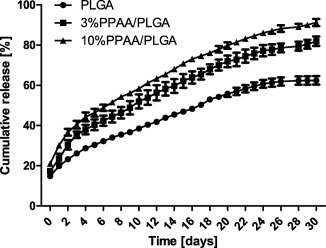
In vitro release profiles of ovalbumin from MPs. In vitro cumulative release profile of OVA from PLGA (•), 3%PPAA/PLGA (▪), and 10%PPAA/PLGA (▲) MPs during 30‐day incubation at 37°C in PBS buffer (pH 7.4). Data are presented as a percentage of the total amount of OVA initially encapsulated in *t* microparticles. Shown are mean ± *SEM* based on *n* = 3 for each MP formulation

The in vitro release kinetics from the three microparticle formulations was examined in PBS for 30 days. Briefly, particles were suspended in PBS and incubated at 37°C under constant agitation. Samples were centrifuged and supernatant collected daily. A burst release was observed during the initial phase with a cumulative percentage of 14.8 ± 0.3% for the PLGA only, 17.4 ± 0.8% for the 3% PPAA/PLGA blend, and 21.0 ± 1.2% for the 10% PPAA/PLGA blend. On completion of the 30‐day incubation, total cumulative ovalbumin released from MPs was 62.4 ± 2.1% for PLGA, 81.9 ± 2.4% for 3% PPAA/PLGA blend, and 91.2 ± 1.8% for 10% PPAA/PLGA blend. However, the complete release of the theoretical maximum encapsulated OVA in the three types of MPs was not achieved over the course of the study (Figure 3).

### 3.2 PPAA‐mediated endosomal disruption

**Figure 4:**
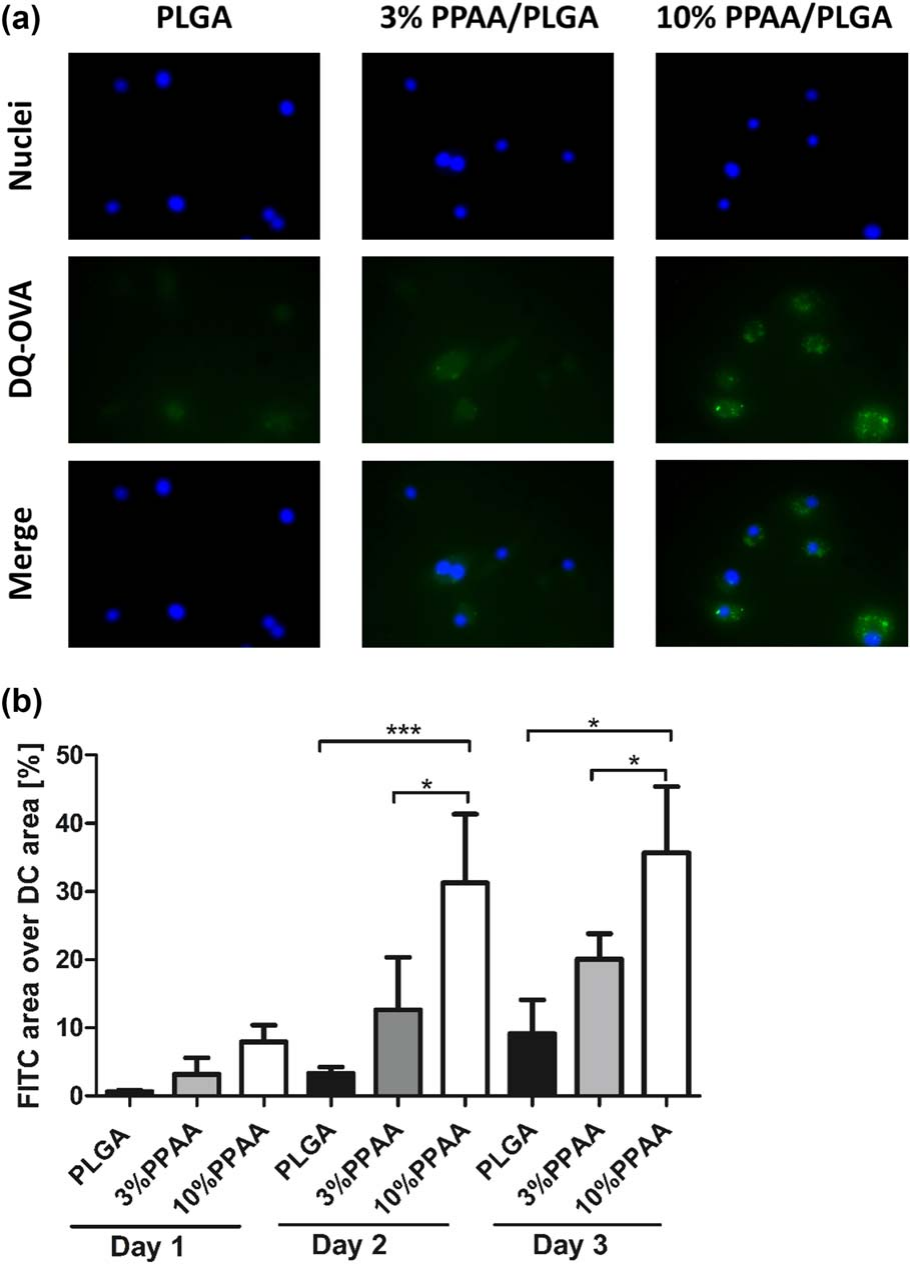
MP‐mediated endosomal disruption. Endosomal disruption was analyzed by microscopy. (a) Representative microscope images of DCs with DQ‐OVA adsorbed MPs, PLGA (left), 3%PPAA/PLGA blend (middle), and 10%PPAA/PLGA blend (right) from day 2 of 3 day test period. Top, blue stained cell nuclei. Middle, green labeled DQ‐OVA MPs. Bottom, merged images of cell nuclei, and DQ‐OVA MPs. Scale bar, 20 μm. (b) Microscopy‐based quantification of microparticles escaped from endosome to cytosol of DCs. Data are presented as mean ± *SEM* for *n* =3. Values of *p* (where *<.05 and ***<.001) were calculated using a two‐way ANOVA with a Bonferroni post‐test

The PPAA‐mediated endosomal escape was examined by microscopy using DQ‐OVA, a fluorogenic protease substrate. A strong fluorescence quenching effect is observed when proteins are heavily labeled with BODIPY dyes. On hydrolysis of DQ‐OVA to single, dye‐labeled peptides by proteases, quenching is relieved, producing brightly fluorescent products. After incubation with DCs for 3 days, DQ‐OVA labeled particles became detectable in the cytosol, determined by diffused fluorescence, versus endosomes, established by punctate florescence patterns^14^ (Figure 4a). The intensity of cytosolic localization markedly increased for all three microparticle formulations over the course of the study (Figure 4b). Interestingly, the 10% PPAA/PLGA blend MPs showed promoted cytosolic localization in the first day with 7.9 ± 2.5% FITC area/DC area, compared to 3.2 ± 2.4%, and 0.6 ± 0.2% for the 3% blend and the PLGA only formulations, respectively. Although PLGA only MPs were also able to escape the endosome to some degree, a significant increase was observed with both the 3 and 10% blend. The capacity to disrupt endosomal membranes increased directly proportional to the percentage of PPAA in the particle formulation.

### 3.3 Antigen‐specific T cell proliferation study

**Figure 5:**
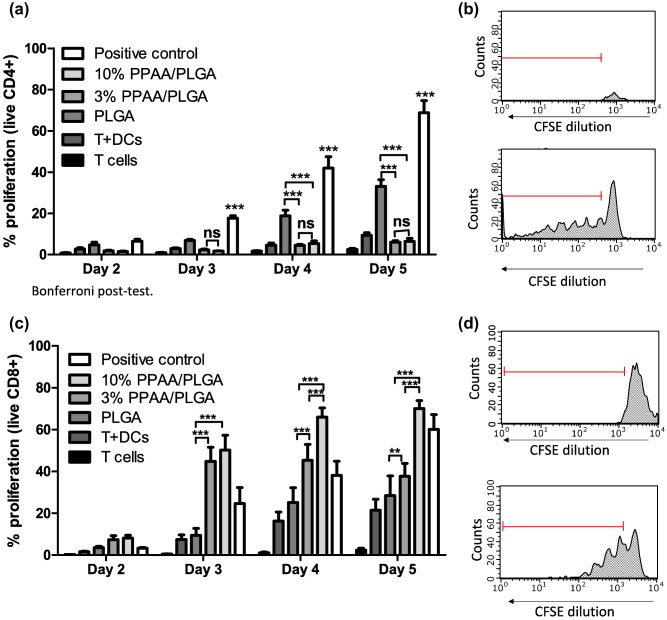
Flow cytometric analysis of OVA specific carboxyfluorescein succinimidyl ester (CFSE) stained T‐cell proliferation assay stimulated MPs‐treated BMDCs. (a) Murine bone marrow derived DCs were incubated with PPAA/PLGA MPs, PLGA MPs for 24 hr or pulsed with OVA peptide 323–339 for 3 hr as a positive control. DCs where then washed and co‐cultured with CD4+ CFSE labeled T cells isolated from OT‐II mice for 5 days. Each day cells were pulled, live/dead, and CD4 stained and proliferation assessed via CFSE dilution by flow cytometry. (b) Representative flow gating scheme of negative (top) and positive (bottom) controls shown. (c) DCs were incubated with polymer blend and PLGA only MPs for 24 hr or pulsed with OVA peptide 257–264 for 3 hr as a positive control. DCs were washed and co‐cultured with CD8+ T CFSE labeled T cells isolated from OT‐I mice for 5 days. Cells were pulled every day, live/dead, and CD8 stained and proliferation addressed through CFSE dilution by flow cytometry. (d) Representative flow cytometry gating scheme for negative (top) and positive (bottom) controls

DCs pretreated for 24 hr with OVA‐loaded microparticle formulations were co‐cultured with CFSE‐labeled OVA‐specific T cells for 5 days. As shown in Figure 5a,b, DCs incubated with PLGA only MPs triggered significantly higher CD4^+^ T cell proliferation beginning on day 4 compared to 3% PPAA/PLGA blend and 10% PPAA/PLGA blend. However, neither of the two PPAA blends promoted CD4^+^ T cell proliferation during the 5‐day study. These results demonstrate PLGA MPs primarily target delivery to endosomes and promote MHC Class II presentation. In contrast, DCs treated with 3% PPAA/PLGA blend MPs and 10% PPAA/PLGA blend MPs triggered significantly elevated CD8^+^ T cell proliferation compared to PLGA MPs, beginning on the day 3 (Figure 5c,d), whereas thePLGA MP was no different from the negative controls. These results demonstrate OVA encapsulated in PPAA/PLGA MPs is effectively delivered to the cytosol, enabling loading, and presentation on MHC‐I to activate CD8^+^ T cells.

## 4 DISCUSSION

This study has provided a novel approach to endosomal escape strategies, enabling the tuning of antigen delivery to either endosomal or cytosolic compartments to shape subsequent CD4^+^ and CD8^+^ T cell responses. Particles of approximately 1 µm in size were fabricated using PPAA/PLGA polymer blends, and were effectively phagocytosed by murine bone marrow derived DCs. The PPAA component allowed for disruption of endosomes, leading to the release of cargo into the cytosol and presentation of antigen on MHC Class I molecules. MHC‐I presentation by DCs was confirmed by the activation of CD8^+^ T cells in a manner directly proportional to the amount of PPAA present in the blend.

Enhancing endosomal escape of endocytosed therapeutic protein antigens can increase the efficacy of protein vaccines through increased activation of cytotoxic T lymphocytes. These CD8^+^ T cells are capable of specifically recognizing malignant cells or cells infected with pathogens, leading to the activation and proliferation of effector cells which then exert cytotoxic functions to eliminate compromised cells. As such, various mechanism for endosomal membrane disruption have been proposed in the last decade, including strategies utilizing ultrasound,^15^, ^16^, ^17^ enzymatic degradation,^18^, ^19^, ^20^ pH,^21^, ^22^ and light.^23^ In addition to these efforts, biodegradable polymeric delivery systems have attracted significant attention due to their loading capability, low toxicity, and ease of use. A number of studies have shown that particle size, particle charge, and delivery site have an impact on uptake and immune stimulation.^24^, ^25^, ^26^, ^27^ Nanometer‐scale particles can be taken up through pinocytosis, a mechanism not exclusive to APCs, whereas particles larger than ∼7 µm are not efficiently engulfed, and the cargo released in the extracellular space.^28^ These studies, in combination with previous work in our lab,^10^, ^29^, ^30^, ^31^, ^32^ guided the design criteria of the microparticle formulations. The polymer blends investigated herein consisted of PPAA, a pH responsive anionic polymer, and PLGA, a well characterized polymer. Microparticles were fabricated using a modified double emulsion water‐oil‐water (w/o/w) solvent extraction method. PLGA not only acted as a hydrophobic core that facilitated the formation of the blend particles, but also minimized degradation by serving as the structural support for the encapsulation of antigen. Fabricated MPs incorporating PPAA maintained similar size and morphology, with only small shifts in surface charge, and loading amounts.

Application of biodegradable MPs as an antigen delivery system depends on the ability to release payload in a controlled fashion. All three formulations demonstrated an initial burst followed by a steady release phase of encapsulated antigen. The 10% PPAA/PLGA blend MPs demonstrated a modestly faster and higher cumulative release, where the rate of release was directly proportional to the PPAA content. Previous work by Acuña et al. demonstrated electrostatic repulsion between oxygen atoms in ether groups of poly(ethylene oxide) and bovine serum albumin^33^ a weak polyelectrolyte and modestly negatively charged protein. Work by Weijers et al. demonstrating a stronger negative charge of OVA^34^ plus the increased polarity of carboxyl groups, compared to ether, lead us to believe a strong repulsion between the two is possible. Although not tested here, we hypothesize the negatively charged carboxyl groups on PPAA lead to a charge repulsion‐based faster release of the negatively charged encapsulated OVA protein.

The ability of PPAA to promote endosomal escape of loaded antigen was examined using murine bone marrow derived DCs. Dendritic cells incubated with PLGA MPs showed a punctuate pattern of fluorescence indicating localization within endosomal compartments. In contrast, the extent of cytosolic distribution dramatically increased in cells incubated with PPAA/PLGA MPs. While the precise mode of endosomal escape was not addressed here, previous work has demonstrated the protonation of PPAA carboxylate ions into deionized carboxylic acid groups at lower pH values encountered during endosomal trafficking causes PPAA to partition into membranes, disrupting the lamellar structure, and releasing internalized material.^35^, ^36^, ^37^, ^38^, ^39^, ^40^ Lastly, DCs treated with all three microparticle formulations were co‐cultured with antigen‐specific T cells to assess activation of MHC‐I versus MHC‐II loading, and stimulation of the cognate T cell subset. Results demonstrate PLGA MPs are promote delivery of antigen to endosomal compartments activating CD4^+^ T cells. Notably, MPs containing PPAA efficiently promoted delivery of antigen to the cytosol, loading onto MHC Class I molecules and presentation to CD8^+^ T cells, at levels comparable to the positive control.

## 5 CONCLUSIONS

Particulate vaccine carriers have been extensively reported in recent years. Despite decades of research, endosomal escape for cytosolic delivery of antigen in APCs for subsequent activation of cytotoxic T cells is still considered a bottleneck in the design of efficacious vaccines. This study utilized a biodegradable polymeric blend carrier composed of PLGA and PPAA, a pH sensitive membrane destabilizing polymer, to induce endosomal escape and target cytosolic delivery of antigen. We demonstrated microparticles fabricated of PPAA/PLGA blends maintained high encapsulated amounts of OVA and showed similar release kinetics to MPs fabricated of PLGA only. In addition, PPAA/PLGA blend MPs were able to target cytosolic delivery of cargo, whereas PLGA MPs remained primarily in the endosome. This was demonstrated through co‐cultures of DCs treated with MPs with OVA‐specific transgenic T cells. CD4+ T cells only proliferated when activated by DCs treated with OVA‐loaded PLGA microparticles, whereas CD8+ T cells became activated only in response to DCs treated with OVA‐loaded PPAA/PLGA microparticle blends. This work indicates that blends of PPAA incorporated into PLGA as a controlled release delivery system maintain the ease of fabrication and loading versatility of PLGA, while enabling tunable CD4^+^ and CD8^+^ T cell responses to improve design of new vaccines.
